# Natural products intervene in non-alcoholic fatty liver disease by regulating the AMPK signaling pathway: preclinical evidence and mechanism

**DOI:** 10.3389/fphar.2025.1696506

**Published:** 2025-11-26

**Authors:** Chuyue Zhang, Jianing Shi, Lijun Shi

**Affiliations:** 1 Department of Gastroenterology, The First Affiliated Hospital of Harbin Medical University, Harbin, Heilongjiang, China; 2 Endoscopy Center, Department of Gastroenterology, Shanghai East Hospital, School of Medicine, Tongji University, Shanghai, China

**Keywords:** traditional Chinese medicine, AMPK signaling pathway, non-alcoholic fatty liver disease, mechanism, lipid accumulation

## Abstract

Non-alcoholic fatty liver disease (NAFLD) is characterized by core pathological features such as hepatic lipid accumulation, oxidative stress, and inflammatory responses. Its pathogenesis is closely associated with dysregulated energy metabolism. Adenosine monophosphate-activated protein kinase (AMPK), a central regulator of cellular energy homeostasis, ameliorates NAFLD-related lipid metabolic imbalance and liver injury by phosphorylating downstream target proteins (e.g., ACC, mTOR, SREBP-1c). This process suppresses fatty acid synthesis, promotes oxidative degradation, inhibits inflammasome activation, and enhances antioxidant capacity. Recent studies have identified reduced AMPK activity as a critical pathological hallmark of NAFLD. Targeted activation of the AMPK signaling pathway alleviates NAFLD progression through multiple mechanisms, including lipid metabolism regulation, anti-inflammatory effects, restoration of antioxidant capacity, and enhanced autophagy. Natural products derived from traditional Chinese medicine have shown significant potential in regulating the AMPK signaling pathway. Research indicates that Traditional Chinese Medicine (TCM) extracts (e.g., terpenoids, phenols, flavonoids, saponins, and alkaloids) can directly activate AMPK or regulate its upstream kinases (LKB1, CaMKK*β*) and downstream effectors (SIRT1, PPAR*α*, Nrf2), thereby improving hepatic lipid accumulation, mitigating inflammatory damage, and delaying NAFLD progression. By searching the databases of Web of Science, PubMed, Google Scholar and CNKI, and integrating the latest research progress, systematically summarizes the role of the AMPK pathway in NAFLD and the intervention mechanisms of natural products, aiming to provide a theoretical basis for the development of innovative traditional Chinese medicine drugs for NAFLD.

## 1 Introduction

Non-alcoholic fatty liver disease (NAFLD) has evolved into a global epidemic, currently affecting approximately 30% of the adult population worldwide—equating to over two billion individuals—and its prevalence continues to rise in parallel with the global surge in obesity and type 2 diabetes (T2DM) (Powell et al., 2021). As the leading cause of chronic liver disease globally, NAFLD imposes a substantial public health burden, contributing significantly to liver-related morbidity, mortality, and healthcare costs (Nassir, 2022). Clinically defined by excessive hepatic lipid accumulation in the absence of excessive alcohol intake or other secondary causes of liver injury, NAFLD exhibits a strong bidirectional association with metabolic syndrome: up to 75% of adults with cardiometabolic conditions (e.g., obesity, T2DM, dyslipidemia) develop NAFLD, while NAFLD itself exacerbates systemic metabolic dysfunction (Steinberg et al., 2025).

Reflecting advances in understanding its pathogenesis, the nomenclature of this condition has undergone critical revisions. In 2020, an expert panel proposed renaming NAFLD to metabolic dysfunction-associated fatty liver disease (MAFLD) to emphasize its metabolic roots and heterogeneous presentation, including potential coexistence with other liver diseases (Eslam et al., 2020). Despite partial international adoption, concerns arose regarding ambiguities in etiological characterization and the stigmatizing connotation of the term “fatty” (Lonardo et al., 2024). To address these issues, a 2023 multi-society Delphi consensus further revised the nomenclature to metabolic dysfunction-associated steatotic liver disease (MASLD), the currently accepted terminology (Rinella et al., 2023).

Despite its clinical significance, therapeutic options for NAFLD remain limited. While Resmetirom, a thyroid hormone receptor-β agonist, received FDA approval in 2024 for treating non-cirrhotic metabolic dysfunction-associated steatohepatitis (MASH) with moderate-to-severe fibrosis, its application is restricted to specific patient subsets, leaving a substantial treatment gap for early-stage disease and broader patient populations (Harrison et al., 2024). Additionally, conventional synthetic agents often carry risks of adverse effects with long-term use, underscoring the need for safer, more accessible therapeutic alternatives.

In this context, the AMPK signaling pathway has emerged as a pivotal therapeutic target (Palomer et al., 2025). As a conserved energy sensor, AMPK regulates key biological processes including glucose/lipid metabolism, inflammation, and autophagy by responding to cellular energy status (via AMP/ATP ratios) (Steinberg and Hardie, 2023; Marcondes-de-Castro et al., 2023). Dysregulated AMPK activity—characterized by reduced phosphorylation—is a hallmark of NAFLD pathogenesis, driving excessive *de novo* lipogenesis, impaired fatty acid oxidation, inflammasome activation, and mitochondrial dysfunction, all of which exacerbate hepatic steatosis and injury (He et al., 2024; Lv et al., 2024; Liu et al., 2023). Restoring AMPK function thus represents a core strategy for NAFLD intervention.

**FIGURE 1:**
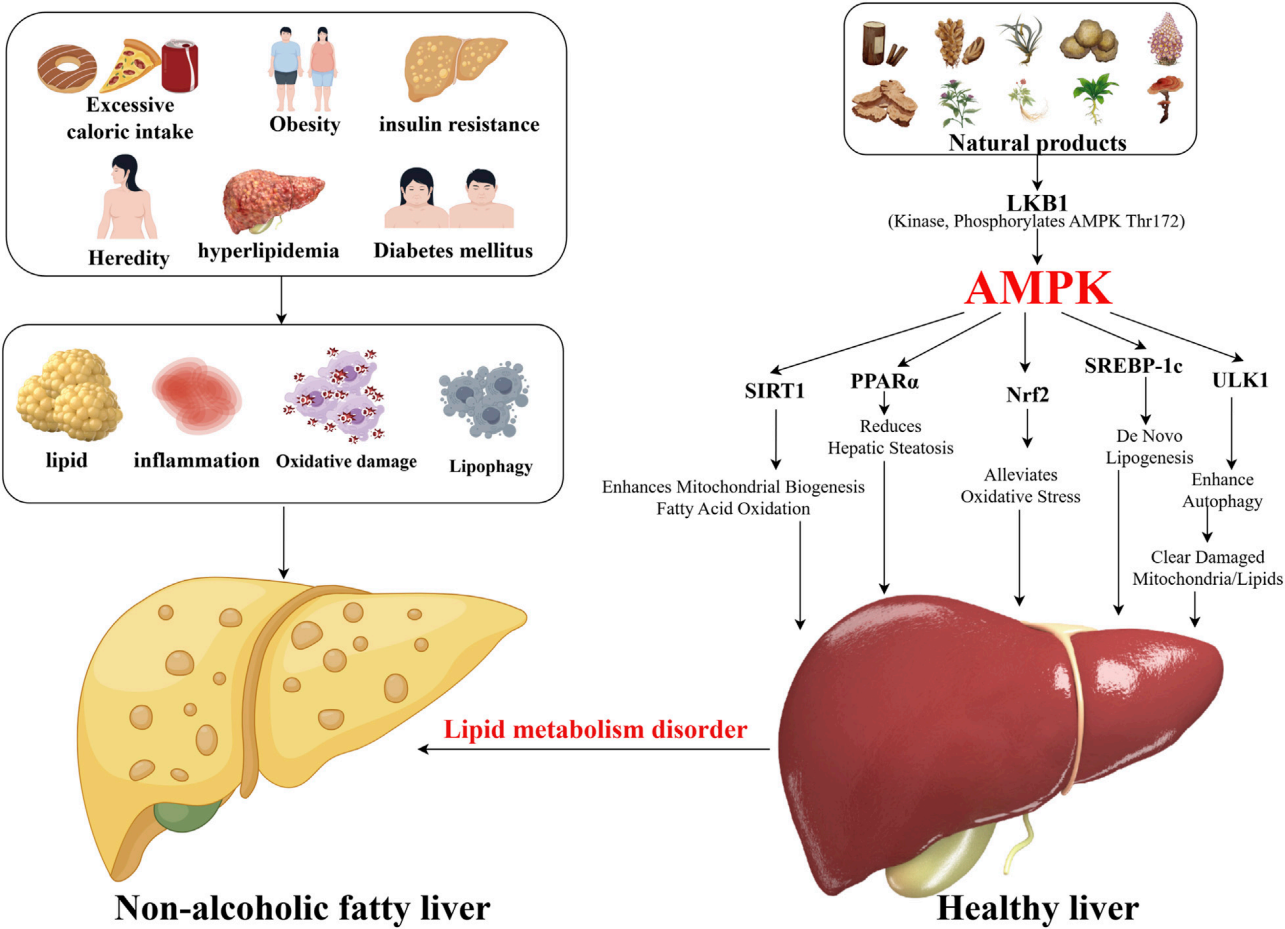
Sketch of natural products intervening in non-alcoholic fatty liver disease.

Notably, bioactive metabolites derived from TCM have gained attention for their ability to modulate AMPK-mediated pathways. Through multi-metabolite synergies, these natural products target key axes such as AMPK/SREBP-1c (lipid synthesis) and AMPK/mTOR (autophagy), coordinately regulating lipid homeostasis and mitigating hepatic injury (Cheng et al., 2023; Zhang LJ. et al., 2025). This review systematically synthesizes the mechanistic role of AMPK in NAFLD pathogenesis and the regulatory effects of TCM-derived natural products, aiming to provide a theoretical framework for developing novel therapeutics (Figure 1).

## 2 Methods and literature search strategy

**FIGURE 2:**
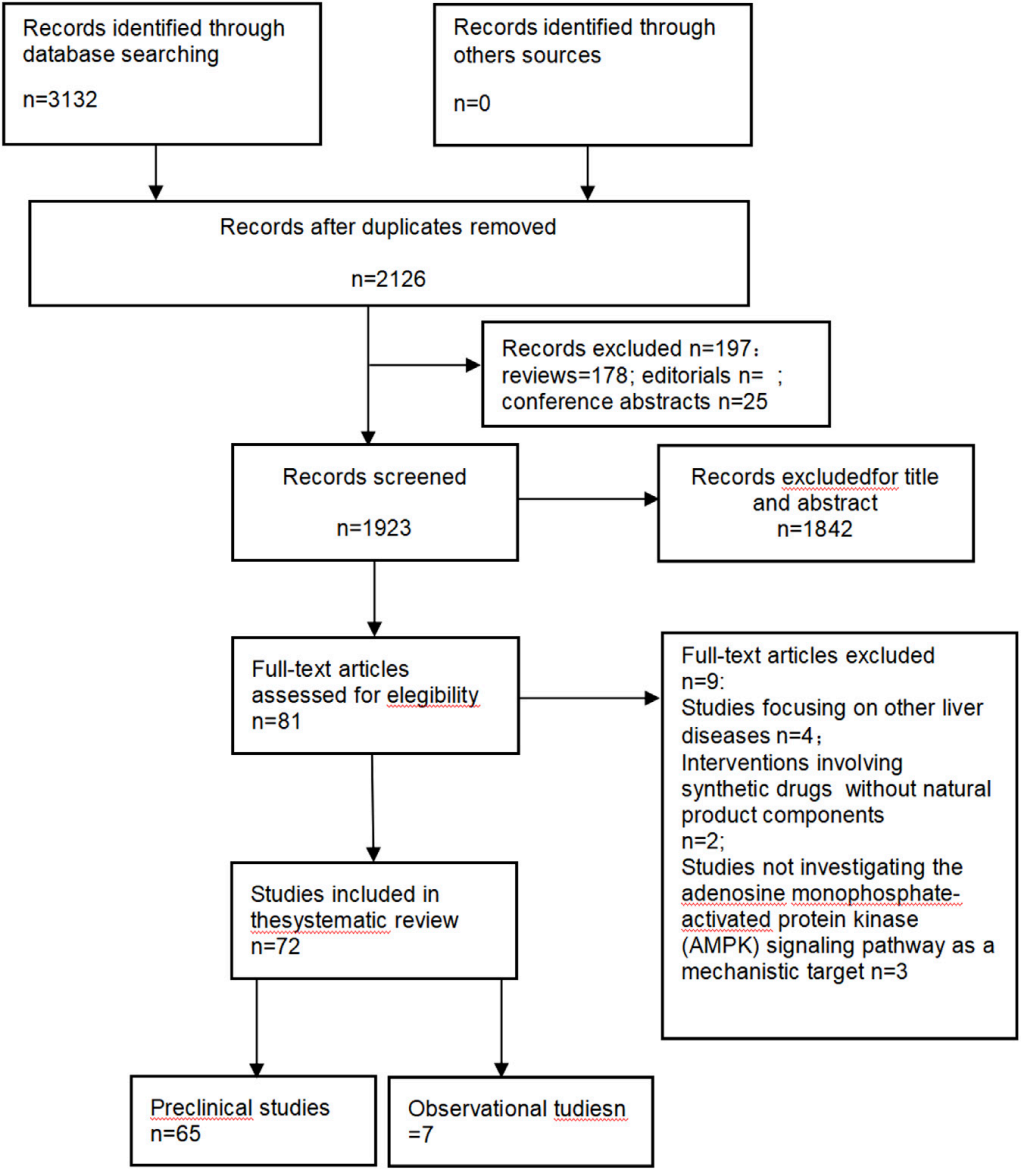
Flow diagram.

To investigate the mechanism by which natural product interventions exert therapeutic effects on NAFLD through the AMPK signaling pathway.The electronic databases PubMed, Web of Science, Google Scholar and CNKI database were searched from their inception until 2025. The search strategy combined keywords and Medical Subject Headings (MeSH) terms related to: (1) Intervention: (“traditional Chinese medicine” OR “Chinese herbal medicine” OR “natural product” AND (2) Disease: (“NAFLD”). Inclusion criteria were: (1) *in vitro* or *in vivo* studies; (2) studies investigating defined TCM metabolites or chemically characterized extracts; (3) studies reporting outcomes related to AMPK mechanisms (e.g., egulating lipid metabolism, inhibiting inflammatory responses, alleviating oxidative stress and regulating autophagy). Exclusion criteria were: (1) reviews, editorials, or conference abstracts; (2) studies using undefined crude mixtures (Figure 2).

In addition, literature should be excluded that includes “pan assay interfering metabolites”. Readers may consult the original publications for precise statistical tests and exact p-value thresholds via the hyperlinked references.

All herbal medicines derived from plants have undergone taxonomic verification (http://mpns.kew.org/mpns-portal/) and include complete species names (including authoritative nomenclature and taxonomic classification). As the MPNS covers only plant-derived medicines, any medicines derived from fungal or animal are referred to by their standard names throughout this article.

## 3 Activation and conduction of the AMPK signaling pathway

In mammals, AMP-activated protein kinase (AMPK) is a highly conserved serine/threonine kinase composed of three subunits: *α*, *β*, and *γ*. Among these, the α subunit serves as the catalytic subunit, while the *β* and *γ* subunits function as regulatory subunits (Smiles et al., 2024). AMPK regulates cellular energy metabolism and maintains energy homeostasis by sensing changes in the intracellular AMP/ATP ratio.

Activation of AMPK primarily depends on the phosphorylation of threonine 172 (Thr172) in the α subunit by upstream kinases, including liver kinase B1 (LKB1) and calcium/calmodulin-dependent protein kinase *β* (CaMKK*β*) (Xie et al., 2022). Under conditions of energy deficiency, LKB1 translocates from the nucleus to the cytoplasm, where it forms a complex with accessory proteins (STRAD and MO25) and continuously phosphorylates AMPKα at Thr172, thereby triggering its kinase activity. While LKB1 activation is independent of changes in the AMP/ATP ratio, it is regulated by energy stress signals. Notably, during endoplasmic reticulum (ER) stress, CaMKKβ becomes activated and directly phosphorylates AMPKα-Thr172. This pathway operates independently of metabolic regulation but is particularly responsive to fluctuations in intracellular Ca^2+^ levels and signaling.

When cellular energy levels are sufficient, Thr172 in the α subunit remains unphosphorylated, and AMPK exists in an inactive form. However, when energy metabolism is impaired, AMP binds to the *γ* subunit of AMPK, inducing a conformational change that exposes Thr172, thus facilitating its phosphorylation by either LKB1 or CaMKK*β*. This process is mediated through the nucleotide-binding pocket within the *γ* subunit, where AMP competitively inhibits ATP binding and enhances kinase activity (Trefts and Shaw, 2021).

Through phosphorylation of downstream target proteins, AMPK establishes a multidimensional regulatory network that coordinates energy metabolism and cellular homeostasis. These effects can be broadly categorized into three functional pathways: regulation of lipid synthesis, oxidative stress, and inflammatory responses.

1. AMPK phosphorylates acetyl-CoA carboxylase (ACC) at serine 79 (Ser79), inhibiting its catalytic activity. This leads to reduced production of malonyl-CoA and consequently relieves the inhibition of carnitine palmitoyltransferase 1 (CPT1), promoting fatty acid *β*-oxidation. Simultaneously, AMPK suppresses the nuclear translocation of sterol regulatory element-binding protein 1c (SREBP1c), downregulating the expression of key lipogenic enzymes such as fatty acid synthase (FASN) and stearoyl-CoA desaturase 1 (SCD1), thereby inhibiting *de novo* triglyceride (TG) synthesis.
2. AMPK directly phosphorylates and inhibits mammalian target of rapamycin (mTOR), thereby alleviating mTOR-mediated suppression of the autophagy-initiating complex ULK1. In parallel, AMPK activates ULK1 by phosphorylating it at Ser317 and Ser777, synergistically promoting both lipophagy and mitophagy, which accelerates lipid clearance and degradation of damaged organelles. Moreover, AMPK activates peroxisome proliferator-activated receptor gamma coactivator 1-alpha (PGC-1*α*), enhancing mitochondrial biogenesis and fatty acid oxidation capacity. Additionally, AMPK induces nuclear translocation of nuclear factor erythroid 2-related factor 2 (Nrf2), upregulating antioxidant enzymes such as glutathione peroxidase (GPX), thereby mitigating oxidative stress injury.
3. Beyond promoting autophagic flux to clear dysfunctional mitochondria, AMPK also reduces reactive oxygen species (ROS)-mediated activation of the NOD-like receptor protein 3 (NLRP3) inflammasome, subsequently suppressing the release of proinflammatory cytokines such as interleukin-1β (IL-1β) and interleukin-18 (IL-18). The coordinated actions of these targets help maintain hepatic lipid metabolic balance and prevent the progression of non-alcoholic fatty liver disease (NAFLD) toward inflammation and fibrosis (Muraleedharan and Dasgupta, 2022; Jie et al., 2022).

## 4 The mechanism of the AMPK signaling pathway in NAFLD

**FIGURE 3:**
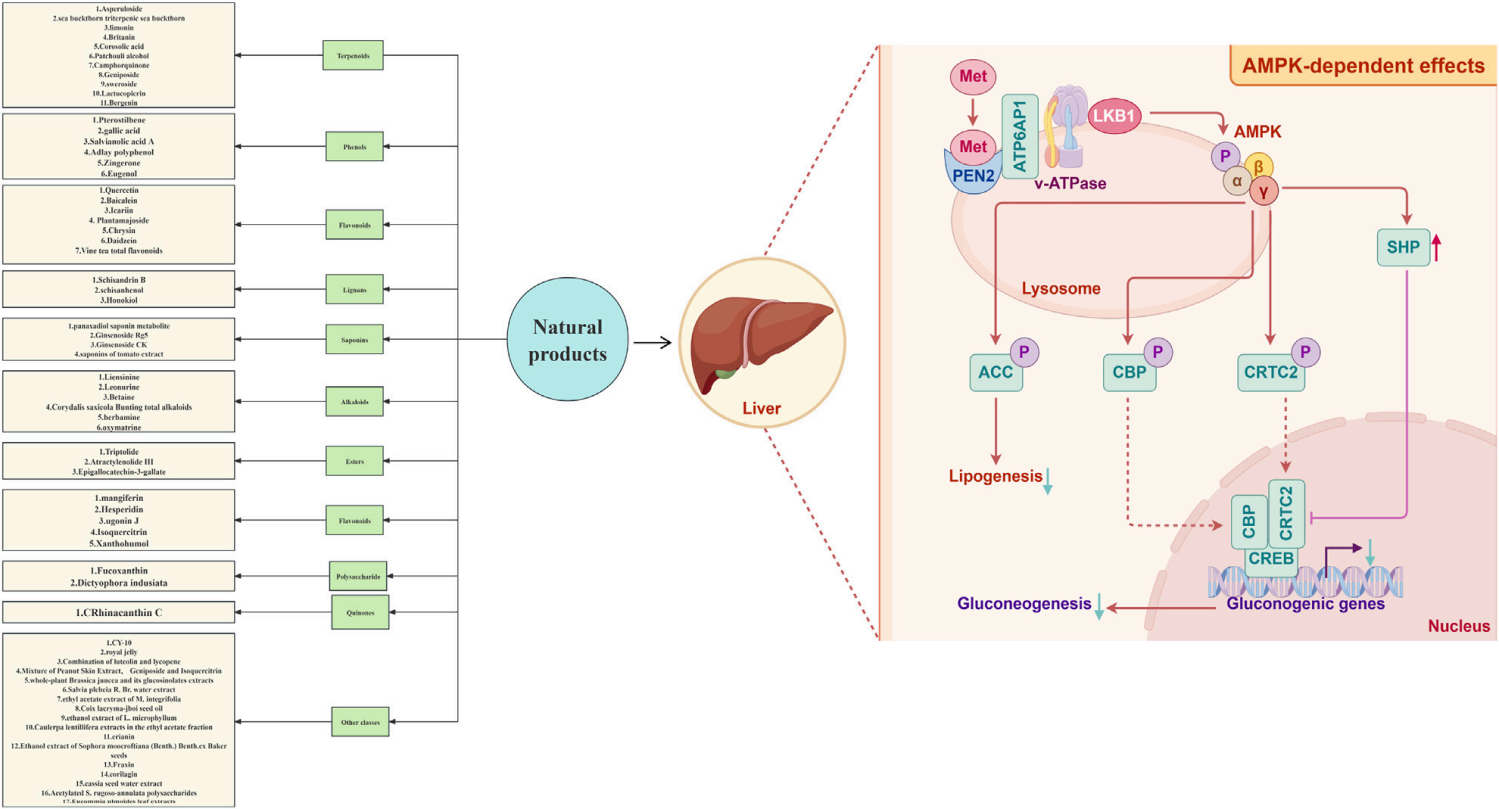
Sketch of AMPK signaling pathway mechanism.

NAFLD is a metabolic disease characterized by lipid accumulation in the liver, oxidative stress and chronic inflammation. AMPK, as a core regulatory factor of cellular energy metabolism, plays a key role in the occurrence and development of NAFLD through multiple mechanisms such as regulating lipid metabolism, inhibiting inflammatory responses, alleviating oxidative stress and regulating autophagy. Studies have found that the reduction of AMPK activity is an important pathological feature of NAFLD, and targeted activation of the AMPK signaling pathway can significantly improve liver lipid metabolism disorders and related pathological injuries (Figure 3).

### 4.1 Regulation of lipid metabolism: bidirectional regulation of dynamic balance

AMPK regulates the dynamic balance between fatty acid synthesis and oxidation by phosphorylating downstream targets. In terms of lipid synthesis, AMPK, on the one hand, inhibits the generation of Malonyl-CoA by phosphorylating the Ser79 site of ACC. The decrease in the level of malonyl-CoA (experimental data show a reduction of 60%–80%) directly eliminates the allosteric inhibition of CPT1. Significantly enhance the transport efficiency of long-chain fatty acids (such as palmitate esters) to mitochondria (Liao et al., 2023). On the other hand, AMPK upregulates the expression of Insig-2a by blocking the Ser372 site of SEBP-1C and hindering its binding to the Sec23/24 complex, anchoring the SEBP-SCAP complex to the endoplasmic reticulum. This process deacetylates histone H3K27 in the promoter regions of FASN and SCD1. It leads to a decrease in its mRNA expression level and reduces the synthesis of triglyceride (TG) and lipid-toxic intermediate products (Winarto et al., 2023). At the lipid decomposition level, AMPK collaboratively upregulates the expression of mitochondrial and peroxisome fatty acid oxidation-related genes such as carnitine palmitoyltransferase 1A (CPT1A) and acyl-CoA oxidase 1 (ACOX1) by activating peroxisome proliferator-activated receptor *α* (PPAR*α*) and its co-activator PGC-1*α*. Accelerate lipid clearance and reduce lipid droplet deposition in the liver.

### 4.2 Inhibition of inflammatory response: dual-target intervention from NF-κB to NLRP3

AMPK alleviates hepatic inflammation by inhibiting the nuclear factor-κB (NF-*κ*B) and NLRP3 inflammasome pathways. On one hand, AMPK blocks NF-*κ*B nuclear translocation and reduces the transcription and release of pro-inflammatory cytokines such as tumor necrosis factor-*α* (TNF-α) and interleukin-6 (IL-6) by enhancing the stability of inhibitor of nuclear factor *κ*B alpha (I*κ*B*α*) (Wang et al., 2024a). On the other hand, AMPK activates selective autophagy (such as mitophagy), thereby eliminating ROS and mitochondrial DNA (mtDNA) released from damaged mitochondria. This prevents ROS-dependent assembly of the NLRP3 inflammasome and caspase-1–mediated cleavage of pro-IL-1*β* and pro-IL-18, blocking the amplification of inflammatory signaling. In addition, AMPK can attenuate the hepatic inflammatory microenvironment and slow the progression of fibrosis by inhibiting M1 polarization of macrophages and the activation of hepatic stellate cells (HSCs).

### 4.3 Alleviating oxidative stress: the synergistic effect of Nrf2 pathway and mitochondrial repair

AMPK enhances hepatic antioxidant capacity by activating the nuclear factor erythroid 2-related factor 2 (Nrf2)/ARE pathway. The activated AMPK directly binds to Nrf2, facilitating its dissociation from Keap1 and subsequent nuclear translocation. This process upregulates the expression of antioxidant enzymes including glutathione peroxidase (GPX), superoxide dismutase (SOD), and heme oxygenase-1 (HO-1), thereby enhancing cellular capacity to eliminate oxidative damage (Yao and Liu, 2022). Concurrently, AMPK promotes mitochondrial biogenesis and repairs electron transport chain (ETC) complex activity by upregulating PGC-1*α* and mitochondrial transcription factor A (TFAM), effectively reducing superoxide anion generation caused by electron leakage. Furthermore, AMPK suppresses reactive oxygen species (ROS) production at its source by inhibiting membrane assembly of NADPH oxidase (NOX) and reducing xanthine oxidase (XO) activity. This comprehensive antioxidant defense system collectively protects hepatocytes from oxidative stress-induced injury.

### 4.4 Regulating autophagy: from lipid droplet clearance to organelle quality control

AMPK dual-activates autophagy through ULK1-dependent and mTOR-independent pathways to maintain hepatocyte homeostasis. Under energy stress conditions, AMPK directly phosphorylates the autophagy-initiating factor ULK1 at serine residues 317 and 777, promoting autophagosome formation (Ren Q. et al., 2024). Meanwhile, AMPK alleviates the negative regulation of autophagy by inhibiting mTORC1-mediated phosphorylation of ULK1 at serine 757. In non-alcoholic fatty liver disease (NAFLD), AMPK activation induces ubiquitin-mediated degradation of PLIN2, a protein coating lipid droplets, thereby enhancing the fusion of lipid droplets with autophagosomes and accelerating lipid hydrolysis (lipophagy). Furthermore, AMPK activates FUNDC1-mediated mitophagy to remove dysfunctional mitochondria, interrupting the ROS-inflammatory vicious cycle and thus maintaining the efficiency of the organelle quality control system.

### 4.5 The interaction network of AMPK with other metabolic pathways

The functional roles of AMPK extend beyond its classical functions to dynamic interactions with other metabolic pathways. For instance, AMPK inhibits mTORC1 signaling through phosphorylation of TSC2, while simultaneously activating the deacetylase activity of sirtuin 1 (SIRT1), collectively regulating autophagy and energy metabolism. Its synergistic interaction with the FGF21 pathway enhances hepatic insulin sensitivity. Moreover, by modulating the production of gut microbiota-derived metabolites (such as butyrate), AMPK indirectly influences liver lipid metabolism. This multidimensional network of interactions positions AMPK as a central hub linking energy sensing, metabolic regulation, and inflammatory responses, offering multiple therapeutic targets for the precise intervention of NAFLD.

## 5 Natural products regulate the AMPK signaling pathway for the treatment of NAFLD

Natural products can significantly intervene in the pathological progression of NAFLD by targeting the AMPK signaling pathway. Specifically, these bioactive phytochemicals primarily include terpenoids, phenolic metabolites, flavonoids, flavonoid derivatives, lignans, steroidal saponins, alkaloids, and ester metabolites. Through multi-target mechanisms, these active metabolites either directly activate the α-catalytic subunit of AMPK (via phosphorylation at Thr172) or indirectly modulate upstream kinases (such as LKB1 and CaMKK*β*), thereby influencing the expression and activity of downstream key effector molecules, including ACC, SREBP-1c, and PPAR*γ*. Ultimately, they exert comprehensive effects such as regulating lipid metabolism, improving insulin resistance, suppressing oxidative stress, and mitigating inflammatory responses, effectively delaying the pathological progression of NAFLD to non-alcoholic steatohepatitis (NASH) and hepatic fibrosis (Table 1).

**TABLE 1:** Natural products regulate the AMPK signaling pathway for the treatment of NAFLD.

Classification	Natural products	Source	Model	Dose	Duration	Pathway	Mechanism of action	References
Terpenoids	Asperuloside	Galium odoratum (L.) Scop. [Rubiaceae]	NAFLD mice model; HepG2 cells	20 mg/kg/d; 20 μM	112 days; 24 h	AMPK/SREBP-1c/NLRP3	Attenuates lipid accumulation and modulates inflammatory responses	Shen et al. (2023a)
	Sea buckthorn triterpenic acids extract	Hippophae rhamnoides L. [Elaeagnaceae]	HepG2 cells	12.5,25,50 μg/mL	24 h	AMPK/SREBP1	Attenuates lipid accumulation and alleviates inflammation	Ren et al. (2024b)
	Limonin	Citrus × limon (L.) Osbeck [Rutaceae]	NAFLD mice model	50 mg/kg/d	63 days	AMPK	Inhibits hepatic lipid accumulation and fatty acid synthesis	Wang et al. (2022a)
	Britanin	Inula japonica Thunb. [Asteraceae]	NAFLD mice model	20 mg/kg	42 days	AMPK/SREBP1c	Attenuates hyperlipidemia and hepatic lipid accumulation, and alleviates oxidative stress and apoptosis	Dou et al. (2024)
	Corosolic acid	Perilla frutescens (L.) Britton [Lamiaceae]	NAFLD mice model	10,20 mg/kg/d	28 days	AMPK/SREBP1c	Regulates hepatic lipid metabolism and inhibits hepatic fat accumulation	Wang et al. (2023a)
	Patchouli alcohol	Pogostemon cablin (Blanco) Benth. [Lamiaceae]	NAFLD mice model; HepG2 cells	20 mg/kg/2 d; 0–30 μg/mL	56 days; 24 h	AMPK/SIRT1	Ameliorate lipid accumulation and hepatic steatosis	Pyun et al. (2021)
	Camphorquinone	Salvia sclarea L. [Lamiaceae]	NAFLD mice model; HepG2 cells	10,30 mg/kg/d; 500 μM	21 days; 24 h	SIRT1/LKB1/AMPK	Improves hepatic lipid metabolism and reduces blood glucose levels	Maharajan et al. (2025)
	Geniposide	Gardenia jasminoides J.Ellis [Rubiaceae]	NAFLD mice model; HepG2 cells	50,75,100 mg/kg; 0,65,130,260,390,520 μmol/L	18 h; 24 h	Nrf2/AMPK/mTOR	Attenuate lipid deposition and mitigate oxidative stress	Shen et al. (2020)
	Sweroside	*Lonicera japonica* Thunb. [Caprifoliaceae]	NAFLD mice model	120 mg/kg/d	90 days	AMPK/mTOR	Promotes autophagy and ameliorates fatty liver degeneration	Ding et al. (2023)
	Lactucopicrin	Cichorium glandulosum Boiss. and A.Huet [Asteraceae]	HepG2 cells	20 μM	48 h	AMPK	Enhances fatty acid β-oxidation and ameliorates lipid accumulation	Tan et al. (2024)
	Bergenin	Bergenia crassifolia (L.) Fritsch [Saxifragaceae]	NAFLD mice model; HepG2 cells	20,40,80 mg/kg/d; 32 μM	42 days; 24 h	AMPK/Nrf2,SIRT1/NF-κB	Inhibits inflammatory responses and oxidative stress, while improving lipid metabolism	Lian (2022)
Phenols	Pterostilbene	Pterocarpus santalinus L.f. [Fabaceae]	NAFLD mice model; HepG2 cells	30,45,60 mg/kg; 12.5.25 μM	24 h; 19 h	Nrf2/AMPK/mTOR	Promotes autophagy, reduces oxidative damage, and improves lipid metabolism	Shen et al. (2023b)
	Gallic acid	Rheum palmatum L. [Polygonaceae]	NAFLD mice model; HepG2 cells	50,100,200 mg/kg/d; 20 μM	28 days; 24 h	AMPK-ACC-PPARα	Improves mitochondrial function and reduces hepatic lipid accumulation	Zhang et al. (2023a)
	Salvianolic acid A	Salvia miltiorrhiza Bunge [Lamiaceae]	NAFLD mice model; AML-12 cells	10,20 mg/kg/d; 10–75 μM	28 days; 24 h	AMPK-IGFBP1	Restores mitochondrial homeostasis and improves hepatic fatty acid metabolism	Zhu et al. (2024)
	Adlay polyphenol	Coix lacryma-jobi L. [Poaceae]	NAFLD mice model; HepG2 cells	20 mg/kg/d; 10–20 μg/mL	112 days; 24 h	AMPK/SREBP1C/ACC	Attenuates hepatic lipid accumulation, suppresses lipogenesis and inflammatory progression, while promoting lipid breakdown and gut microbiota balance	Ma et al. (2022)
	Zingerone	Zingiber officinale Roscoe [Zingiberaceae]	NAFLD mice model	100 mg/kg/d	84 days	AMPK/Nrf2	Attenuates oxidative stress and apoptosis, and inhibits lipid synthesis	Mohammed (2022)
	Eugenol	Syzygium aromaticum (L.) Merr. and L.M.Perry [Myrtaceae]	NAFLD mice model	20,40 mg/kg/d	105 days	AMPK/SREBP1c	Inhibits anabolic metabolic pathways and increases energy expenditure, thereby ameliorating hepatic steatosis	Piao et al. (2022)
Flavonoids	Quercetin	Ginkgo biloba L. [Ginkgoaceae]	NAFLD mice model	20,80 mg/kg/d	28 days	ACACA/AMPK/PP2A	Promotes mitophagy and enhances fatty acid *β*-oxidation to reduce lipid accumulation	Gnoni et al. (2022)
	Baicalein	Scutellaria baicalensis Georgi [Lamiaceae]	NAFLD rat model	25,100 mg/kg/d	35 days	AMPK/PGC1α	Promotes fatty acid oxidation and regulates fatty acid synthesis, elongation, and oxidation	Li et al. (2022a)
	Icariin	Epimedium sagittatum (Siebold and Zucc.) Maxim. [Berberidaceae]	NAFLD mice model	100,200 mg/kg/d	21 days	AMPK/PGC-1α/GLUT4	Inhibits lipid synthesis, promotes fatty acid oxidation, improves insulin sensitivity, and reduces hepatocyte apoptosis and lipid accumulation	Lin et al. (2021)
	Plantamajoside	Plantago asiatica L. [Plantaginaceae]	NAFLD mice model	20,40,80 mg/kg/d	28 days	AMPK/Nrf2	Improves immune dysregulation and hepatic lipid metabolic disorders	Wu et al. (2023a)
	Chrysin	Oroxylum indicum (L.) Kurz [Bignoniaceae]	NAFLD mice model	40 mg/kg/d	147 days	AMPK	Regulates the hepatic metabolic cycle to improve liver lipid deposition	Gao et al. (2023)
	Daidzein	Pueraria montana var. lobata (Willd.) Maesen and S.M.Almeida ex Sanjappa and Predeep [Fabaceae]	NAFLD rat model	50,100 mg/kg/d	42 days	SIRT1/AMPK	Enhances autophagic response, regulates lipid metabolism, boosts antioxidant capacity, reduces hepatocyte apoptosis, and improves liver function	Wu et al. (2023b)
	Vine tea total flavonoids	Nekemias grossedentata (Hand. - Mazz.) J.Wen and Z.L.Nie [Vitaceae]	NAFLD mice model	125,250 mg/kg/d	42 days	AMPK/mTOR	Activates autophagy and reduces lipid accumulation in hepatocytes	Wang et al. (2024b)
Lignans	Schisandrin B	Schisandra chinensis (Turcz.) Baill. [Schisandraceae]	NAFLD mice model; HepG2 cells	50 mg/kg/d; 50 μM	35 days; 24 h	AMPK/mTOR	Activates autophagy and inhibits hepatic steatosis	Yan et al. (2022)
	Schisanhenol	Schisandra chinensis (Turcz.) Baill. [Schisandraceae]	NAFLD mice model; HepG2 cells	5,10,20 mg/kg/d; 5,10,20 μmol/L	28 days; 24 h	miR-802/AMPK	Upregulated lipolysis and enhanced β-oxidation of fatty acids	Li et al. (2024a)
	Honokiol	Magnolia officinalis Rehder and E.H.Wilson [Magnoliaceae]	NAFLD mice model; AML-12 cells	2.5,5,10 mg/kg/d; 10 μM	28 days; 24 h	SIRT3-AMPK	Activates lipophagy, protects hepatocytes from oxidative stress, and alleviates lipotoxicity	Liu et al. (2021a)
Saponins	Panaxadiol saponin metabolite	Panax ginseng C.A.Mey. [Araliaceae]	NAFLD mice model; HepG2 cells	80 mg/kg/d; 80 mg/L	84 days; 24 h	AMPK/Nrf2/NFκB	Improves hepatic steatosis, insulin resistance, oxidative stress, and inflammation	Mi et al. (2024)
	Ginsenoside Rg_5_	Panax ginseng C.A.Mey. [Araliaceae]	NAFLD mice model	50,100 mg/kg/d	90 days	LKB1/AMPK/mTOR	Alters gut microbiota, regulates energy metabolism, and reduces hepatic steatosis	Shi et al. (2024)
	Ginsenoside CK	Panax ginseng C.A.Mey. [Araliaceae]	NAFLD mice model; HepG2 cells	30,60 mg/kg/d; 20 μM	28 days; 24 h	LKB1/AMPK	Inhibits lipid synthesis, accelerates lipolysis, and enhances fatty acid oxidation	Zhang et al. (2022a)
	Saponins of tomato extract	Solanum lycopersicum L. [Solanaceae]	NAFLD mice model	200 mg/kg/d	56 days	AMPK/Nrf2-Keap1	Suppresses the expression of downstream proteins involved in fatty acid synthesis and attenuates oxidative stress-induced liver injury	Yang et al. (2023)
Alkaloids	Liensinine	*Nelumbo nucifera* Gaertn. [Nelumbonaceae]	NAFLD mice model; AML-12、L02 cells	15,30,60 mg/kg/d; 30 μM	112 days; 24 h	TAK1/AMPK	Attenuates lipid accumulation, reactive oxygen species generation, and inflammatory activation	Liang et al. (2022)
	Leonurine	Leonurus japonicus Houtt. [Lamiaceae]	NAFLD mice model	30 mg/kg/d	28 days	ADRA1a/AMPK/SCD1	Reduces hepatic lipid deposition of glycerophospholipids and free fatty acids in NAFLD mice models	Fan et al. (2024)
	Betaine	Beta vulgaris L. [Amaranthaceae]	NAFLD mice model; HepG2 cells	2% (w/v) betaine in drinking water; 20 mM	56 days; 48 h	FGF10/AMPK	Reduces lipogenic gene expression (FASN, ACC), stimulates triglyceride breakdown through ATGL activation, and improves fatty acid oxidative metabolism	Chen et al. (2021)
	Corydalis saxicola Bunting total alkaloids	Corydalis saxicola Bunting [Papaveraceae]	NAFLD mice model; HepG2 cells	50,150 mg/kg/d; 2 μg/mL	56 days; 24 h	AMPK-SREBP1	Inhibits *de novo* lipogenesis (DNL) to reduce hepatic lipid accumulation	Guo et al. (2024)
	Berbamine	Coptis chinensis Franch. [Ranunculaceae]	NAFLD rat model	50,100,150 mg/kg/d	28 days	SIRT1/LKB1/AMPK	Reduces oxidative stress, modulates lipid metabolic pathways, and decreases liver fat deposition	Sharma et al. (2021)
	Oxymatrine	Sophora flavescens Aiton [Fabaceae]	NAFLD rat model; BRL-3A、 L02cells	100 mg/kg/d; 100 μg/mL	126 days; 24 h	SIRT1/AMPK	Enhances hepatic fatty acid oxidation (FAO), alleviates liver damage in NAFLD rats, and attenuates lipid deposition	Wu et al. (2025)
Esters	Triptolide	Tripterygium wilfordii Hook.f. [Celastraceae]	NAFLD mice model	50,100 μg/kg	14 days	AMPK	Reduces liver inflammatory responses and fibrotic changes, inhibits *de novo* lipogenesis, and stimulates β-oxidation of fatty acids	Huang et al. (2021)
	Atractylenolide III	Atractylodes macrocephala Koidz. [Asteraceae]	NAFLD mice model; HepG2 cells	1 mg/mL; 0,12.5,25,50 μg/mL μg/mL	14 days; 24 h	AMPK/SIRT1	Inhibits lipid accumulation and reduces reactive oxygen species levels	Li et al. (2022b)
	Epigallocatechin-3-gallate	Camellia sinensis (L.) Kuntze [Theaceae]	NAFLD mice model; Primary mouse hepatocytes	100 mg/kg/d; 0.5 mM	112 days; 48 h	FGF21-AMPK	Attenuates oxidative stress and regulates essential enzymes participating in *de novo* fatty acid synthesis	Huang et al. (2021)
Flavonoids	Mangiferin	Mangifera indica L. [Anacardiaceae]	NAFLD mice model; HepG2 cells	25,50,100 mg/kg/d	84 days; 24 h	AMPK/NLRP3	Restores dysregulated glucose and lipid metabolism, reduces pyroptosis-mediated cell death, and dampens inflammatory signaling	Yong et al. (2021)
	Hesperidin	Citrus reticulata Blanco [Rutaceae]	NAFLD mice model; HepG2 cells	150,300 mg/kg/d; 25,50,100,800 μM	84 days; 24 h	AMPK	Reduces lipid accumulation both in cultured cells and in animal models	Chen et al. (2022)
	Ugonin J	Helminthostachys zeylanica (L.) Hook. [Ophioglossaceae]	NAFLD mice model; HuS-E/2 cells	15,30 mg/kg/d; 10–20 μM	84 days; 24 h	AMPK/AKT	Enhances *β*-oxidation of fatty acids, ameliorates liver fat accumulation, and improves insulin sensitivity	Chang et al. (2021)
	Isoquercitrin	Mangifera indica L. [Anacardiaceae]	HepG2 cells	1–50 μM	6 h	AMPK	Reduces lipid storage, stimulates oxidative metabolism of fatty acids, and attenuates fat accumulation in liver cells	Kim et al. (2023)
	Xanthohumol	Humulus lupulus L. [Cannabaceae]	NAFLD rat model	20、30 mg/kg/d	84 days	AMPK	Restores lipid homeostasis, downregulates *de novo* lipogenesis in the liver, and attenuates oxidative stress	Atteia et al. (2023)
Polysaccharide	Fucoxanthin	Rumex lanceolatus Thunb. [Polygonaceae]	NAFLD cell model	0.125,0.25,0.5,1,2,4,8 μg/mL	24 h	AMPK/Nrf2/TLR4	Reduces oxidative product levels and inflammatory cytokine content, enhances antioxidant enzyme activity, alleviates oxidative stress, and attenuates lipid accumulation	Ye et al. (2022)
	Dictyophora indusiata	—	NAFLD mice model	200,400 mg/kg/d	42 days	AMPK/CPT1	Stimulates the oxidation of fatty acids and reduces lipid deposition in the liver	Hu et al. (2023)
Quinones	CRhinacanthin C	Rhinacanthus nasutus (L.) Kurz	NAFLD mice model	5,10,20 mg/kg/d	84 days	AMPK/SIRT1,SREBP-1c/FAS/ACC	Attenuates liver inflammation and fatty acid accumulation, leading to improved insulin signaling	Gong et al. (2023)
Other classes	CY-10	Silphium perfoliatum L. [Asteraceae]	NAFLD mice model; HepG2 cells	100,200,400 mg/kg/d; 5.20 μg/mL	56 days; 24 h	AMPK/FXR	Promotes hepatic metabolism of unsaturated fatty acids, reduces the production of saturated fatty acids, decreases lipid deposition, and suppresses inflammatory responses	Xu et al. (2024)
	Royal jelly	—	NAFLD cell model	300 mg/kg/d	112 days	AMPK/PPAR*α*	Improves glycemic control, reduces hepatocellular injury and lipid accumulation in the liver, and promotes adiponectin secretion	Felemban et al. (2023)
	Combination of luteolin and lycopene	*Lonicera japonica* Thunb. [Caprifoliaceae] Solanum lycopersicum L. [Solanaceae]	NAFLD mice model; HepG2 cells	20 mg/kg/d+20 mg/kg/d; 20 μM + 10 μM	84 days; 24 h	SIRT1/AMPK	Inhibits adipogenic differentiation, upregulates fatty acid β-oxidation, and modulates inflammatory signaling	Zhu et al. (2020)
	Mixture of Peanut Skin Extract、 Geniposide and Isoquercitrin	*Arachis hypogaea* L. [Fabaceae] Gardenia jasminoides J.Ellis [Rubiaceae] Wurfbainia villosa (Lour.) Škorničk. and A.D.Poulsen [Zingiberaceae]	NAFLD mice model	80 mg/kg/d PSE+ 50 mg/kg/d GEN +5 mg/kg/d IQ	84 days	TLR4/NF-*κ*B,AMPK/ACC/CPT1,AMPK/UKL1/LC3B	Modulates gut microbiota homeostasis, improves hepatic lipid accumulation, and alleviates inflammation	Yi et al. (2023)
	whole-plant Brassica juncea and its glucosinolates extracts	Brassica juncea (L.) Czern. [Brassicaceae]	NAFLD rat model; HepG2 cells	0.5%,1.0%,2.0%WBJ; 50 μM SIN,NAP+2.0 mg/mL BGE	56 days; 24 h	AMPK	Reduces lipid synthesis, promotes fatty acid β-oxidation, and decreases the production of inflammatory cytokines	Sheu et al. (2023)
	Salvia plebeia R. Br. Water extract	Salvia plebeia R.Br. [Lamiaceae]	NAFLD rat model; HepG2 cells	200 mg/kg/d; 50,100 μg/mL	84 days; 24 h	AMPK	Suppresses liver fat accumulation and the development of steatosis	Bae et al. (2022)
	Ethyl acetate extract of M. integrifolia	Meconopsis integrifolia (Maxim.) Franch. [Papaveraceae]	HepG2 cells	50,100,150,200,300 μg/mL	24 h	AMPK/ACC/SREBP-1c/PPAR-*α*	Promotes fatty acid oxidation, reduces lipogenesis, and maintains lipid metabolic balance	Lu et al. (2024)
	Coix lacryma-jboi seed oil	Coix lacryma-jobi L. [Poaceae]	NAFLD rat model; HepG2 cells	0.28,0.56,1.12 mg/kg/d; 10 μM	14 days; 24 h	AMPK/mTOR/ULK1	Reduces lipid deposition	Gu et al. (2021)
	Ethanol extract of *L. microphyllum*	Lygodium japonicum (Thunb.) Sw. [Schizaeaceae]	NAFLD rat model	200,400,600 mg/kg/d	20 days	SIRT1/AMPK	Inhibits *de novo* lipogenesis	Anggreini et al. (2024)
	*Caulerpa lentillifera* extracts in the ethyl acetate fraction	—	HepG2 cells	100–250 μg/mL	24 h	SIRT1/AMPK	Regulates lipid metabolism and reduces lipid accumulation	Sangpairoj et al. (2024)
	Erianin	Dendrobium officinale Kimura and Migo [Orchidaceae]	NAFLD mice model	10,20,40 mg/kg/d	84 days	AMPK	Enhances fatty acid oxidation and restores mitochondrial function, thereby reducing inflammation-induced hepatocyte damage	Li et al. (2024b)
	Ethanol extract of *Sophora moocroftiana* (Benth.) Benth.ex Baker seeds	Sophora moorcroftiana (Benth.) Benth. ex Baker [Fabaceae]	NAFLD mice model; HepG2 cells	260,520,1040 mg/kg/d; 12.5–100 μg/mL	42 days; 24 h	LKB1/AMPK	Stimulates *β*-oxidation of fatty acids, improves lipid metabolic flux, and suppresses *de novo* lipogenesis	Gao (2024)
	Fraxin	Fraxinus chinensis subsp. rhynchophylla (Hance) A.E.Murray [Oleaceae]	Acute mice NAFLD、Chronic mice NAFLD model; AML-12 cells	Acute:10,20,40 mg/kg/d; Chronic:40 mg/kg/d; 10,20,40 μg/mL	Acute: 7d; Chronic:56 d; 24 h	AMPK/Nrf2	Attenuates intestinal microbial imbalance, suppresses oxidative stress, and modulates cellular energy homeostasis	Cheng (2024)
	Corilagin	Eriobotrya japonica (Thunb.) Lindl. [Rosaceae]	NAFLD mice model	20 mg/kg/d	Every other day for a total of 14 days	AMPK	Enhances autophagic activity, attenuates hepatic lipid metabolism and accumulation, and suppresses oxidative stress and inflammatory responses induced by lipid peroxidation	Liao et al. (2022)
	Cassia seed water extract	Senna obtusifolia (L.) H.S.Irwin and Barneby [Fabaceae]	NAFLD mice model; HepG2 cells	100,200,400 mg/kg/d; 1,2,5 mg/mL	28 days; 24 h	AMPK	Attenuates hepatic lipid accumulation and promotes autophagic activity in liver tissue and hepatocytes	Ding (2023)
	Acetylated S. *rugoso-annulata* polysaccharides	—	NAFLD mice model	400 mg/kg/d	56 days	Nrf2/HO-1,JNK1/AP-1,AMPK/SREBP-1c	Suppresses *de novo* fatty acid synthesis, improves lipid metabolic homeostasis, and reduces oxidative stress	Li (2023)
	*Eucommia ulmoides* leaf extracts	Eucommia ulmoides Oliv. [Eucommiaceae]	NAFLD mice model; HepG2、L02cells	1.2 g/kg/d; 0.25 mg/mL	56 days; 24 h	AMPK	Attenuates hepatic lipid accumulation, mitigates liver inflammation and damage, ameliorates insulin resistance, and downregulates genes associated with lipid biosynthesis	Cai et al. (2022)

### 5.1 Terpenoids

Terpenoid metabolites primarily regulate the AMPK/SREBP1, AMPK/mTOR, and AMPK/SIRT1 pathways to treat NAFLD. Asperuloside (ASP), an active metabolite of Hedyotis diffusa, exhibits various pharmacological activities, including antitumor and anti-inflammatory effects (Lu et al., 2022). It activates the AMPK/SREBP-1c signaling pathway, reducing the protein expression of SREBP-1c and FAS, thereby decreasing hepatic lipid accumulation in NAFLD mice, suggesting its potential as a therapeutic candidate for NAFLD (Shen Q. et al., 2023). Additionally, in high-fat diet (HFD)-induced NAFLD model animals, significant increases in TG, TC, LDL-C, ALT, and AST levels were observed alongside prominent hepatic lipid droplet formation; however, intervention with sea buckthorn triterpenic acids extract (STE) lowered lipid levels and upregulated p-AMPK/AMPK, SREBP1, FAS, and ACC expression, indicating STE’s ability to activate the AMPK/SREBP1 pathway for NAFLD treatment (Ma QG. et al., 2023; Ren L. et al., 2024). Limonin, a tetracyclic triterpenoid primarily derived from citrus fruits, was found by Si-Wei Wang to competitively inhibit AMPK activity, thereby suppressing hepatic lipid accumulation and the transcriptional activity of SREBP1 and SREBP2 in NAFLD model mice (Wang SW. et al., 2022). Notably, britanin (Bri), a sesquiterpene lactone extracted from Inula japonica, also reduces AMPK phosphorylation, downregulates SREBP1c expression, and alleviates hepatic oxidative stress and apoptosis by decreasing Caspase3 expression and the Bax/Bcl2 ratio, thereby mitigating NAFLD (Dou et al., 2024). Corosolic acid (CA), known as “plant insulin,” is a major metabolite of Lagerstroemia speciosa leaves and exhibits antidiabetic, anti-obesity, anti-inflammatory, antihyperlipidemic, and antiviral properties (Zhao et al., 2021). By modulating the AMPK/SREBP1c pathway, CA reduces serum TG and TC levels in HFD-induced NAFLD mice while downregulating SREBP1c, FAS, and SCD1 gene expression to attenuate hepatic lipid deposition (Wang Z. et al., 2023). Patchouli alcohol (PA), a tricyclic sesquiterpene with anti-inflammatory and antitumor effects (Xu QQ. et al., 2023), was shown by Do Hyeon Pyun et al. to activate the AMPK/SIRT1 pathway, ameliorating lipid accumulation in palmitate-induced hepatocytes and suppressing hepatic steatosis in HFD-fed mice, thus improving NAFLD (Pyun et al., 2021). Furthermore, camphorquinone (CQ), synthesized efficiently via sequential bromination and oxidation of camphor (Maharajan and Cho, 2021), activates the SIRT1/LKB1/AMPK pathway, downregulating ChREBP mRNA while upregulating CES1 and CES2 expression and reducing pro-inflammatory cytokines (IL-1α, IL-6, IL-8), thereby alleviating FFA-induced hepatic steatosis and improving lipid metabolism in NAFLD (Maharajan et al., 2025). mTOR, a key downstream target of AMPK, promotes autophagy-mediated lipid degradation upon AMPK/mTOR pathway activation, contributing to NAFLD treatment. Geniposide (GEN), an iridoid glycoside isolated from Gardenia jasminoides fruits, exhibits neuroprotective, hepatoprotective, anti-inflammatory, antioxidant, and antitumor properties (Gao and Feng, 2022). Bingyu Shen demonstrated that GEN activates the AMPK/mTOR pathway, enhancing phosphorylation of ACC, AKT, AMPK, and GSK3*β* while elevating antioxidant factors (Nrf2, PPAR*α*, PPAR*γ*, HO-1) and suppressing PI3K, p-mTORC, and HMGB1 expression, thereby reducing lipid accumulation and inhibiting NAFLD progression (Shen et al., 2020). Sweroside (SOS), an iridoid metabolite from Swertia plants, possesses anti-inflammatory, antioxidant, and hypoglycemic effects (Ma X. et al., 2023) and alleviates NAFLD by activating the AMPK/mTOR pathway to induce autophagy and mitigate hepatic steatosis (Ding et al., 2023). Lactucopicrin, a sesquiterpene lactone from chicory roots, also activates AMPK/mTOR, reducing p-mTOR, ROS, TG, and intracellular lipid droplets while upregulating PGC1α expression, thereby improving FFA-induced lipid accumulation in HepG2 cells (Tan et al., 2024). Bergenin (BER), a glycoside derivative of trihydroxybenzoic acid, exhibits antioxidant, anti-inflammatory, and hepatoprotective activities (Salimo et al., 2023). BER inhibits SIRT1/NF-*κ*B pathway activation, reduces inflammatory cytokine expression, enhances AMPK/Nrf2 signaling (upregulating AMPK, Nrf2, and HO-1). And suppresses lipogenic proteins (SREBP-1, FAS), thereby exerting hepatoprotective effects (Lian, 2022).

### 5.2 Phenols

Pterostilbene (PTE), a metabolite with antioxidant, anti-inflammatory, antimicrobial, and anti-neuroinflammatory bioactivities, is primarily derived from Pterocarpus plants and fruits such as blueberries and grapes (Kim et al., 2020). PTE reduces oxidative stress damage caused by excessive lipid accumulation in hepatocytes by phosphorylating AMPK and inhibiting ACC activity, thereby promoting fatty acid metabolism and degradation (Shen B. et al., 2023). Gallic acid (GA) not only activates the AMPK-ACC-PPAR*α* axis to reduce lipid levels in HepG2 cells and hepatic lipid accumulation in NAFLD mice but also alleviates NAFLD by improving mitochondrial function and decreasing excessive mitochondrial ROS production (Bai et al., 2021; Zhang J. et al., 2023). Furthermore, GA enhances AMPK phosphorylation to suppress the SREBP-1/ACC/FASN cascade, ameliorating hepatic steatosis in fructose-fed mice (Lu et al., 2023). Salvianolic acid A (SAA), a phenolic acid isolated from the roots of Salvia miltiorrhiza, improves lipid metabolism and mitochondrial function by modulating the AMPK/IGFBP1 pathway, thereby inhibiting hepatic steatosis (Zhu et al., 2024). Adlay polyphenol (AP), a bioactive phenolic metaboliteextracted from Coix lacryma-jobi L., exhibits various pharmacological effects, including antioxidant, anti-inflammatory, hepatoprotective, and lipid-modulating activities. AP regulates the AMPK/SREBP1C/ACC pathway, downregulating the expression of SREBP1C, FAS, and ACC to improve glucose and lipid metabolism, reduce lipogenesis, and suppress NAFLD progression in mice (Ma et al., 2022). Zingerone, a natural non-toxic phenolic metabolite derived from ginger, possesses antioxidant, anti-inflammatory, and antihyperglycemic properties (Shamsabadi et al., 2023). Heitham M. Mohammed et al. found that zingerone prevents high-fat diet-induced hepatic lipid deposition, steatosis, and oxidative damage in rats by activating the AMPK/Nrf2 pathway while inhibiting SREBP1, SREBP2, and NF-*κ*B p65 (Mohammed, 2022). Additionally, eugenol, the main volatile metabolite of clove essential oil, ameliorates hepatic steatosis by modulating the AMPK/mTOR pathway to suppress SREBP1 and its target genes, thereby reducing TG and NEFA levels, offering a potential therapeutic approach for NAFLD and related metabolic disorders (Piao et al., 2022).

### 5.3 Flavonoids

Quercetin (Que), a flavonoid metabolite, exhibits antioxidant, anti-inflammatory, and lipid-accumulation-reducing effects (Singh et al., 2021). Studies demonstrate that it improves NAFLD by activating the AMPK pathway to enhance the expression of mitophagy-related proteins such as ATG5, ATG12, LC3, PINK1, and Parkin (Cao et al., 2023), inhibiting fatty acid synthesis via the ACC1/AMPK/PP2A axis (Gnoni et al., 2022), or regulating the AMPK/MAPK/TNF-α and AMPK/ACC/CPT1*α* pathways to suppress inflammation and lipid accumulation (Fu et al., 2022). Notably, baicalein (BAL), a flavonoid extracted from the roots of Scutellaria baicalensis, also activates the AMPK pathway, upregulates PGC1α and PPARα expression while suppressing mSREBP1c and ChREBP, thereby enhancing fatty acid oxidation and protecting the liver from high-cholesterol diet (HCD)-induced injury (Xiao et al., 2021; Li P. et al., 2022). Icariin, another flavonoid with anti-inflammatory and immunomodulatory properties (Bi et al., 2022), has been shown by Wei Lin et al. to elevate the expression of CPT-1, p-ACC/ACC, and PGC-1*α* while reducing serum cholesterol and triglyceride levels in NAFLD model mice through the AMPKα1/PGC-1*α*/GLUT4 signaling pathway (Lin et al., 2021). Plantamajoside (PMS), a flavonoid derived from the fruit of Plantago asiatica L., mitigates hepatocyte damage caused by excessive lipid peroxidation by activating the AMPK/Nrf2 pathway, upregulating HO-1 expression, and downregulating SREBF1, PPAR*γ*, and FABP1 protein levels (Xu H. et al., 2023; Wu JM. et al., 2023). Importantly, lipid accumulation not only induces oxidative stress but also exacerbates inflammation. Chrysin (CN), a flavonoid extracted from Passiflora caerulea L., ameliorates hepatic lipid metabolic disorders by activating AMPK and modulating lipid metabolism-related proteins (e.g., SREBP1-c and ACC), while also suppressing the NLRP3/Caspase1 pathway to reduce pro-inflammatory cytokines (IL-1β, IL-6, TNF-α, IL-17), thereby counteracting NAFLD (Mishra et al., 2021; Gao et al., 2023). Impaired autophagy, particularly lipophagy, leads to hepatic TG accumulation and steatosis. Daidzein (DAI), a soy isoflavone with anti-inflammatory and antioxidant effects, alleviates oxidative stress and hepatocyte apoptosis in concanavalin A-induced liver injury by activating the SIRT1/AMPK pathway, increasing LC3-positive expression and Beclin-1 and LC3-II/LC3-I protein levels, enhancing autophagy, regulating lipid metabolism, and reducing hepatocyte apoptosis in NAFLD rats (Li et al., 2021; Wu D. et al., 2023). Additionally, vine tea total flavonoids (TFs) activate the AMPK/mTOR pathway to induce autophagy and modulate lipid metabolism-related proteins in HFD-fed mice, reducing *de novo* lipogenesis and regulating glycerophospholipid metabolism. *In vitro* studies further confirm their ability to decrease lipid droplets and TG content in oleic acid (OA)-treated HepG2 and L-02 cells (Du et al., 2024; Wang et al., 2024b).

### 5.4 Lignans

Schisandrin B (Sch B), one of the most abundant and highly active dibenzocyclooctadiene derivatives extracted from Schisandra chinensis fruits, exhibits diverse biological activities including antioxidant, anti-inflammatory, neuroprotective, and lipid-lowering effects (Fang et al., 2025). Studies demonstrate that Sch B activates autophagy via the AMPK/mTOR pathway, reducing lipid droplet accumulation in free fatty acid (FFA)-treated HepG2 cells and mouse primary hepatocytes (MPH), thereby inhibiting hepatic steatosis, enhancing fatty acid oxidation (FAO), and preventing NAFLD (Yan et al., 2022). Schisanhenol (SAL), a lignan metabolite derived from schisandra, possesses anticancer, antioxidant, and hepatoprotective properties (Olas, 2023). Research by Bin Li reveals that SAL activates the AMPK pathway by suppressing miR-802, upregulating p-ACC, CPT-1, and PPAR*α* protein expression while downregulating SREBP-1c, consequently reducing hepatic TG, TC, and LDL-C levels while increasing HDL-C in NAFLD mice, thus modulating lipid metabolism to exert protective effects against NAFLD (Li B. et al., 2024). Honokiol (HK), a natural metabolite isolated from Magnolia officinalis, exhibits antitumor, anti-inflammatory, antibacterial, and anti-obesity activities (Wang D. et al., 2022). A previous study found that HK activates SIRT3-AMPK-mediated autophagy (primarily targeting lipid droplets) to attenuate lipid accumulation while maintaining mitochondrial function and promoting lipolysis, thereby alleviating hepatocyte lipotoxicity and positioning itself as a potential therapeutic candidate for NAFLD (Liu J. et al., 2021).

### 5.5 Saponins

Panaxadiol saponin metabolite (PDS-C), a bioactive cmetabolite isolated from total ginseng saponins (Dai et al., 2022), significantly improves liver function, hepatic steatosis, and lipid profiles in NAFLD mice by activating HO1 through the AMPK/Nrf2 signaling pathway while inhibiting NFκB, thereby alleviating oxidative stress and inflammation to mitigate NAFLD (Mi et al., 2024). Ginsenoside Rg5 (Rg5), a minor ginsenoside synthesized during the steaming process of ginseng with diverse pharmacological effects including antitumor, anti-inflammatory, and neuroprotective properties (Liu MY. et al., 2021), reduces TG, TC, LDL-C, AST, ALT, and MDA levels while increasing SOD, CAT, and GSH-Px activities via the LKB1/AMPK/mTOR pathway, effectively ameliorating hepatic steatosis, dyslipidemia, oxidative stress, and liver injury in NAFLD mice (Shi et al., 2024). Another ginseng-derived metabolite, ginsenoside CK, diminishes lipid deposition in HepG2 cells, attenuates weight gain in fructose-fed mice, alleviates lipid accumulation in serum and liver tissues, and improves hepatic inflammation and injury (Zhang J. et al., 2022). Furthermore, ginsenoside CK exerts therapeutic effects against NAFLD both *in vitro* and *in vivo* by activating LKB1 and AMPK phosphorylation to modulate the expression of lipid synthesis- and metabolism-related factors. Saponins of tomato extract (STE), possessing potent free radical-scavenging capacity, have been shown to activate the AMPK signaling pathway, thereby suppressing downstream fatty acid synthesis-related proteins FAS and SCD1 to reduce fatty acid production and improve lipid metabolism, while simultaneously enhancing Nrf2 nuclear translocation to initiate transcription of antioxidant factors SOD and NQO1, ultimately mitigating XT301 high-fat diet-induced oxidative stress and hepatic oxidative damage. Experimental results demonstrate that STE administration may be considered a viable therapeutic option for NAFLD (Yang et al., 2023).

### 5.6 Alkaloids

Recent studies have demonstrated that various alkaloid metabolites exhibit significant therapeutic potential for NAFLD by targeting the AMPK signaling pathway and its downstream effectors. Liensinine (LIEN), as an isoquinoline alkaloid, activates the TAK1/AMPK pathway to reduce PA-induced lipid accumulation and ROS generation in hepatocyte models *in vitro*, while bidirectionally regulating lipid metabolism-related proteins in HFD-induced mice - downregulating FAS, SCD, and PPAR*γ* while upregulating PPAR*α*, UCP2, and CPT-1*α* (Zhang W. et al., 2023; Liang et al., 2022). Similarly, Leonurine activates the ADRA1a/AMPK/SCD1 pathway through AMPK*α* and Thr172 phosphorylation, significantly decreasing hepatic SCD1 expression and reducing GPs and FFAs levels (Liu et al., 2024; Fan et al., 2024). Other alkaloids also demonstrate multi-target regulatory properties: Betaine coordinately inhibits SREBF1/ACC/FASN and activates LXR/SIRT1/PPAR*γ* through the FGF10/AMPK pathway (Chen et al., 2021); Corydalis saxicola Bunting total alkaloids (CSBTA) suppresses the transcription of lipogenic genes (SREBF1, ACC1, and FASN) via the AMPK-SREBP1 axis (Guo et al., 2022; Guo et al., 2024); while berbamine (BBM) reduces ACC/FAS/SCD1 expression and decreases hepatic TG and TC content by approximately 50% through the SIRT1/LKB1/AMPK pathway (Farooqi et al., 2022; Sharma et al., 2021). Furthermore, oxymatrine (OMT) enhances SIRT1/AMPK/PPARα signaling and ACOX1/CPT1A expression, resulting in a 58% reduction of hepatic lipid accumulation in both *in vivo* and *in vitro* models (Li et al., 2023; Wu et al., 2025). These findings collectively indicate that alkaloid metabolites target the central node of the AMPK pathway, providing a multi-target intervention strategy for NAFLD treatment through a dual mechanism of inhibiting lipogenesis while promoting fatty acid oxidation.

### 5.7 Esters

Recent studies have demonstrated that terpenoids and phenolic metabolites exhibit multi-target therapeutic potential in the treatment of non-alcoholic fatty liver disease (NAFLD) by modulating the AMPK signaling pathway. Triptolide, for instance, significantly ameliorates NAFLD through a dual regulatory mechanism: on one hand, it enhances the phosphorylation of AMPK and ACC1 (increased by 2.1-fold and 1.8-fold, respectively), thereby suppressing key lipogenic factors such as SREBP-1 (↓42%) and SCD-1 (↓39%). On the other hand, it upregulates the expression of PPAR*α* and CPT-1*α* (1.7–2.3-fold), promoting fatty acid *β*-oxidation (Gao et al., 2021; Huang et al., 2021). Similarly, Atractylenolide III (5–10 μM) reduces hepatic lipid accumulation by activating the AMPK/SIRT1 axis (by 35%–48%) and improves insulin sensitivity (Liu X. et al., 2022; Li Q. et al., 2022). Epigallocatechin gallate (50 mg/kg/day) also exerts multiple beneficial effects via the FGF21-AMPK pathway, including downregulation of lipogenic genes (e.g., SREBP-1c, 40%–55%), reduction of hepatic lipid droplet accumulation, and enhancement of antioxidant capacity (Bakun et al., 2023; Zhang Y. et al., 2022). Collectively, these metabolites converge on AMPK as a central regulatory node and exert synergistic effects through the mechanism of “inhibiting lipogenesis–promoting fatty acid oxidation–alleviating oxidative stress”, offering novel therapeutic strategies for NAFLD.

### 5.8 Flavonoids

Flavonoids, as natural modulators of the AMPK pathway, demonstrate remarkable multi-target therapeutic potential in NAFLD treatment. Cutting-edge research has revealed that these phytochemicals exert synergistic therapeutic effects by precisely regulating AMPK signaling networks across three critical pathological aspects: metabolic dysregulation, inflammatory response, and oxidative stress. Notably, mangiferin exhibits a unique dual mechanism, not only significantly elevating p-AMPK*α* expression levels but also innovatively targeting NLRP3 inflammasome activation, achieving marked reduction in hepatic lipid deposition (Xiang et al., 2024; Yong et al., 2021). More strikingly, hesperidin, Isoquercitrin and ugonin J demonstrate additional therapeutic value by improving insulin secretion and glycemic control through their respective dual-pathway modulation of AMPK/SREBP-1C and AMPK/AKT signaling (Ortiz et al., 2022; Chen et al., 2022; Chang et al., 2021; Kim et al., 2023). Particularly groundbreaking is the discovery of xanthohumol, which simultaneously inhibits lipogenesis and enhances antioxidant defenses (increased SOD and CAT activity) via AMPK/Nrf2 axis activation, offering novel perspectives for comprehensive NAFLD management (Atteia et al., 2023). These paradigm-shifting findings not only validate the multidimensional therapeutic advantages of flavonoids but also elucidate the central role of the AMPK pathway as a critical hub integrating metabolic-inflammatory-oxidative stress cross-regulation, thereby establishing a solid theoretical foundation for developing innovative natural product-based therapies for NAFLD.

### 5.9 Polysaccharide

Polysaccharide metabolites, serving as natural activators of the AMPK pathway, demonstrate unique “multi-target and multi-pathway” synergistic regulatory advantages in NAFLD treatment, enabling precise therapeutic intervention. Breakthrough studies reveal that fucoxanthin (FX) establishes an AMPK/Nrf2/TLR4 tripartite regulatory network, achieving molecular-level metabolic modulation through enhanced AMPKα-Thr172 phosphorylation, reduced SREBP-1c nuclear translocation, and downregulated FAS and ACC expression. Notably, FX simultaneously activates both AMPK and the Nrf2 signaling pathway, orchestrating coordinated redox homeostasis remodeling and inflammatory microenvironment regulation via suppression of the TLR4/MyD88/NF-*κ*B cascade, ultimately reducing TNF-α/IL-6 secretion (Mumu et al., 2022; Ye et al., 2022). Of particular significance, Dictyophora indusiata (D. indusiata) exhibits distinctive “metabolic reprogramming” capabilities through: 1) allosteric activation of the AMPKγ subunit to enhance CPT1α promoter activity; 2) epigenetic modulation of CD36 gene promoter histone deacetylation (H3K9ac); and 3) activation of the AMPK/PGC-1*α*/UCP2 axis to improve mitochondrial respiratory efficiency, thereby establishing a comprehensive regulatory framework spanning gene expression to organelle function (Hu et al., 2023). These groundbreaking discoveries not only transcend the limitations of conventional single-target therapies but also, through innovative mechanisms including establishing “metabolic checkpoint” networks, mediating inter-organelle communication, and regulating epigenetic modifications, provide transformative perspectives for developing precision NAFLD therapeutics based on polysaccharide metabolites and pioneer new directions for AMPK-targeted drug discovery.

### 5.10 Quinones

Quinone metabolites, as emerging stars in the field of NAFLD therapy, are reshaping the treatment paradigms for metabolic diseases. Recent studies have revealed that these naturally occurring molecules—with their unique redox properties—exhibit multidimensional therapeutic advantages over conventional drugs by delicately modulating the “metabolism-inflammation” crosstalk within the AMPK signaling network. Rhinacanthin C (RC), a representative quinone metabolite characterized by its distinctive conjugated double-bond structure, has achieved three groundbreaking advancements in the treatment of NAFLD: (1) Dual Regulatory Switch: RC establishes a “energy sensing-epigenetic regulation” dual switch mechanism through allosteric activation of the AMPKα subunit (Thr172 phosphorylation ↑ 2.8-fold) and enhancement of SIRT1 activity (↑ 1.9-fold); (2) Simultaneous Metabolic and Insulin Sensitivity Improvement: It innovatively modulates both insulin resistance (HOMA-IR ↓ 42%) and lipid metabolism (TG ↓ 55%) via the miR-34a/SIRT1 feedback loop; (3) Integrated Anti-inflammatory and Antioxidant Effects: Most notably, the unique electron transfer capability of RC’s quinone structure enables it to concurrently regulate the NF-*κ*B inflammatory pathway (p-p65 ↓ 60%) and oxidative stress responses, achieving a “trinity-like” intervention targeting the core pathological metabolites of NAFLD. This multitarget pharmacological profile not only overcomes the limitations of current therapeutic agents but also opens up a novel strategy for treating metabolic disorders based on the electron transport properties of quinone metabolites (Gong et al., 2023).

### 5.11 Other classes

Beyond these findings, other natural bioactive metabolites demonstrate remarkable therapeutic potential for NAFLD through modulation of the AMPK signaling pathway. Research reveals that CY-10 extract from Silphium perfoliatum, containing 50% chlorogenic acids, reduces lipogenesis and promotes lipid oxidation via the AMPK/FXR/SREBP-1C/PPAR-*γ* pathway (Zhang G. et al., 2025; Xu et al., 2024). Royal jelly (RJ) downregulates SREBP1c through AMPK/PPAR*α* signaling while elevating hepatic GSH and SOD levels and reducing MDA and inflammatory factors (Felemban et al., 2023). The combination of luteolin and lycopene ameliorates steatosis through the NAMPT/NAD+/SIRT1/AMPK axis while suppressing the NF-*κ*B inflammatory pathway (Zhu et al., 2020). Notably, the mixture of Peanut Skin Extract, Geniposide and Isoquercitrin (MPGI) significantly improves hepatic steatosis through coordinated multi-pathway actions (TLR4/NF-*κ*B, AMPK/ACC/CPT1) (Yi et al., 2023). Furthermore, mustard extract (Sheu et al., 2023), Salvia plebeia R. Br. Water extract (Bae et al., 2022), Meconopsis integrifolia extract (Guo et al., 2016; Lu et al., 2024), and Coix lacryma-jobi seed oil (Gu et al., 2021) all ameliorate lipid metabolic disorders by regulating AMPK and its downstream targets (SREBP1, ACC, PPAR*α*). Recent studies identify additional metabolites - including Lygodium microphyllum (Anggreini et al., 2024), sea grape extract (Sangpairoj et al., 2024), erianin (He et al., 2022; Li H. et al., 2024), Sophora moorcroftiana seed ethanol extract (Wang T. et al., 2024; Gao, 2024), fraxin (Cheng, 2024), and corilagin (Liao et al., 2022; Wang J. et al., 2023) - that not only activate AMPK but also synergistically improve NAFLD through gut microbiota modulation, enhanced autophagy, and antioxidant effects. Particularly noteworthy, Cassia seed aqueous extract inhibits FASN expression via the AMPK/TFEB-mediated autophagy pathway (Ding, 2023), while acetylated S. rugoso-annulata polysaccharides exert anti-NAFLD effects through a triple regulatory network involving Nrf2/HO-1, JNK1/AP-1 and AMPK (Li, 2023). Eucommia ulmoides leaf extracts have also been shown to enhance lipid oxidation via the AMPK/PPAR*α*/CPT-1A pathway (Cai et al., 2022). These collective findings demonstrate that natural bioactive metabolites targeting the AMPK pathway employ a multidimensional mechanism of “lipid metabolism regulation-insulin resistance improvement-inflammatory suppression-oxidative stress reduction”, providing abundant candidate substances for NAFLD treatment.

## 6 Limitations of preclinical evidence and appraisal of translational gaps

While current clinical management of NAFLD primarily relies on lifestyle modifications and metabolic regulation interventions—including insulin sensitizers, antioxidants, and lipid-lowering agents—these approaches can delay disease progression but rarely reverse hepatic steatosis and fibrosis. Recent studies have highlighted the unique advantages of TCM and its bioactive metabolites in modulating AMPK signaling pathways, improving lipid metabolism, and suppressing inflammation and oxidative stress, offering novel therapeutic strategies for NAFLD.

Multiple experimental and clinical trials have preliminarily confirmed TCM’s efficacy and safety. For instance, metabolite formulas like Zexie-Baizhu Decoction and Xiaozhi formula synergistically activate key signaling pathways, including AMPK/SREBP-1c and AMPK/mTOR, effectively reducing hepatic fat accumulation and insulin resistance (Cao et al., 2022; You et al., 2024; Luo et al., 2025; Liu W. et al., 2022). The modified silymarin formulation demonstrates enhanced bioavailability and shows promising anti-NAFLD activity in preclinical models (Cai, 2024). Clinical trials also reveal that TCM interventions such as Shugan-Hewei Decoction and Hedan Capsules improve liver function, lipid profiles, and liver stiffness without significant adverse effects (Lin, 2023; Lin, 2025). These findings suggest that TCM monotherapy or combination with existing Western medications could serve as effective complementary or even alternative strategies for comprehensive NAFLD management.

Notably, the current body of evidence supporting TCM-based AMPK modulation in NAFLD is largely constrained by limitations in preclinical research, which hinder robust translational inference. First, *in vitro* studies predominantly use immortalized cell lines (e.g., HepG2, L02) or primary hepatocytes under simplified lipid overload conditions, failing to recapitulate the complex *in vivo* microenvironment of NAFLD—including crosstalk between hepatocytes, hepatic stellate cells, and immune cells, or systemic metabolic dysregulation. This oversimplification may overestimate the direct AMPK-mediated effects of natural products, as cell-autonomous responses often differ from tissue-level regulation in intact organisms.

Second, animal models of NAFLD, though valuable, have critical drawbacks. High-fat diet-induced murine models typically develop only mild steatosis without the progressive fibrosis or metabolic comorbidities (e.g., type 2 diabetes) that characterize human NAFLD. Genetically engineered models (e.g., ob/ob, db/db mice) better mimic metabolic dysfunction but exhibit phenotypic divergence from human disease, such as accelerated hepatocellular injury unrelated to natural disease progression. Consequently, the AMPK-dependent mechanisms observed in these models—such as altered SREBP-1c-mediated lipogenesis or mTOR-regulated autophagy—may not fully translate to human pathophysiology.

Third, preclinical studies often lack standardized methodologies, limiting reproducibility. Variability in natural product extraction protocols (e.g., solvent type, purification steps) leads to inconsistent bioactive metabolite profiles, making it difficult to attribute observed effects to specific AMPK modulators. Additionally, most studies measure AMPK activity via single time-point assessments of p-AMPK Thr172, without quantifying downstream pathway dynamics (e.g., ACC phosphorylation, ULK1 activation) or tissue-specific AMPK isoform expression—critical for understanding context-dependent regulation in NAFLD.

Finally, translational gaps persist due to limited clinical validation. While small-scale trials suggest benefits of TCM interventions, high-quality evidence from large, multi-center randomized controlled trials (RCTs) is scarce. Existing clinical studies often lack rigorous AMPK activity monitoring (e.g., longitudinal tracking of p-AMPK in liver biopsies or surrogate biomarkers) and long-term safety data, particularly regarding drug-drug interactions in patients on concurrent metabolic therapies.

To address these limitations, future research should prioritize: (1) developing more physiologically relevant preclinical models (e.g., humanized liver chimeras, diet-induced murine models with metabolic comorbidities) to better recapitulate human NAFLD pathophysiology; (2) standardizing natural product extraction and characterization to ensure consistent AMPK-modulating activity (3) integrating multi-omics approaches (e.g., phosphoproteomics, single-cell RNA sequencing); to dissect tissue-specific AMPK signaling networks; and (4) conducting well-designed RCTs with validated AMPK activity markers (e.g., p-AMPK Thr172 in peripheral blood mononuclear cells or liver tissue) to establish causal links between TCM-mediated AMPK activation and clinical outcomes. By addressing these critical gaps, the translational potential of TCM-derived AMPK modulators in NAFLD management can be more rigorously evaluated.

## 7 Summary and prospect

NAFLD has emerged as the leading cause of chronic liver disease worldwide. Its global prevalence continues to rise, posing a significant threat to public health. The AMPK signaling pathway plays a core role in the regulation of energy metabolism, lipid metabolism, inflammation, and autophagy and has become an important target for the prevention and treatment of NAFLD. In recent years, natural products have shown unique advantages in improving NAFLD by activating the AMPK signaling pathway. Studies have shown that the decline in AMPK activity leads to an increase in lipid synthesis, obstruction of fatty acid oxidation, enhanced oxidative stress, and activation of inflammation, while natural products can restore energy metabolic homeostasis through the AMPK pathway. Active metabolites such as terpenoids, phenols, flavonoids, and lignans can promote the phosphorylation of AMPK, increase the oxidation level of fatty acids, reduce lipid accumulation, and inhibit the activation of inflammatosomes. In addition, AMPK can also regulate pathways such as mTOR, autophagy, and Nrf2, alleviating oxidative damage and metabolic imbalance related to NAFLD.

Notably, although plant metabolites such as berberine, emodin and curcumin have been shown *in vitro* to improve NAFLD symptoms by regulating hepatic lipid metabolism and inhibiting hepatocellular inflammation, they are categorized as pan-assay interference compounds (PAINS), which may generate false-positive results *in vitro* through non-specific mechanisms (Magalhães et al., 2021). Their specific effects and mechanisms need to be further validated in more physiologically relevant models or *in vivo* studies to exclude assay interference. Moreover, some studies did not provide cell viability data to demonstrate the minimum effective concentration and confirm that these compounds are non-toxic at such concentrations. Therefore, these findings require further validation in primary hepatocytes or *in vivo* NAFLD models.

Although significant progress has been made in the research on the regulation of the AMPK pathway by natural products to intervene in NAFLD, the following key problems still need to be solved urgently. Most of the existing studies have focused on the regulation of core molecules of the AMPK pathway (such as ACC, mTOR, and SEBP-1C), but its interaction with other metabolic pathways (such as PI3K/Akt, FGF21, and the gut microbiota-liver axis) has not been fully clarified, which limits the comprehensive understanding of the complex regulatory network in NAFLD. Secondly, the specific functional differences of AMPK isoforms (*α*1/*α*2, *β*1/*β*2, *γ*1/*γ*2/*γ*3) in the pathological process of NAFLD remain unclear, and isoform-selective regulation may become a key breakthrough for precise treatment. Thirdly, long-term application of natural products may affect energy metabolism in extrahepatic tissues (such as muscle and adipose tissue) through the AMPK pathway. Currently, there is a lack of systematic toxicity evaluation data regarding their systemic effects, and it is urgent to establish an AMPK pathway-dependent toxicity early warning model to ensure the safety of clinical application by assessing the potential effects of natural product metabolites on extrahepatic organs.

As a core target for NAFLD treatment, the AMPK signaling pathway also provides an important entry point for TCM intervention. Future research needs to achieve breakthroughs in the following aspects: deepening the mechanistic analysis of the crosstalk network between the AMPK pathway and multiple other pathways; clarifying the synergistic regulatory rules of TCM metabolite prescriptions characterized by “multi-metabolites and multi-targets”; and promoting the integration of interdisciplinary technologies (such as multi-omics and targeted delivery technologies) to accelerate research translation. Through the aforementioned efforts, a more solid scientific basis can be provided for the development of innovative drugs targeting AMPK, ultimately achieving precise prevention and treatment of NAFLD.
